# Predictors of nest growth: diminishing returns for subordinates in the paper wasp *Polistes dominula*

**DOI:** 10.1007/s00265-018-2502-x

**Published:** 2018-05-11

**Authors:** Lena Grinsted, Jeremy Field

**Affiliations:** 1 School of Life Sciences, University of Sussex, John Maynard Smith Building, Falmer, Brighton, BN1 9QG UK; 2 Present Address: School of Biological Sciences, Royal Holloway University of London, Egham, Surrey, TW20 0EX UK; 3 Present Address: Centre for Ecology and Conservation, University of Exeter, Penryn Campus, Cornwall, TR10 9EZ UK

**Keywords:** Social insects, Michener’s paradox, Cooperation, Social evolution, Altruism, Group living

## Abstract

**Abstract:**

In cooperative breeders, subordinates that have alternative reproductive options are expected to stay and help dominant breeders only as long as they contribute to group productivity, if their fitness is linked with colony success. Female *Polistes dominula* paper wasps live as cooperative breeders in small groups of typically fewer than 10 females. Subordinates tend to have high-quality outside options, and so could choose alternative breeding tactics if their work efforts increased productivity negligibly. In the founding stage before workers emerge, we tested the effect of various predictors on nest growth, as a proxy for group productivity, and explored the shape of the relationship between group size and nest growth. We found group size to be the only significant predictor of nest growth: variation among body sizes within the group showed no effect, suggesting a lack of size-dependent task specialization in this species. Average body size and average genetic relatedness between group members similarly showed no effects on nest growth. Group size had a non-linear effect so that per-capita benefits to nest growth decreased in larger groups, and groups of 10 or more would benefit negligibly from additional group members. Hence, females might be better off pursuing other options than joining a large group. This finding helps to explain why *P. dominula* groups are usually relatively small in our study population. Further studies may illuminate the mechanisms behind the smaller per-capita nest growth that we found in larger groups.

**Significance statement:**

Identifying which factors influence the productivity of animal groups is key to understanding why different species breed cooperatively in groups of varying sizes. In the paper wasp *Polistes dominula*, we investigated the growth rate of nests as a measure of group productivity. We found that average body size, the variation in body sizes within the group, and average genetic relatedness between group members did not affect nest growth, while group size had a strong, positive effect: nests grew faster with more group members, but the per-capita benefit decreased in larger groups. The addition of extra group members in groups of 10 or more had negligible effects on nest growth. Hence, wasps may be better off pursuing other options than joining large groups. This finding helps to explain why groups normally consist of fewer than 10 wasps in this population.

**Electronic supplementary material:**

The online version of this article (10.1007/s00265-018-2502-x) contains supplementary material, which is available to authorized users.

## Introduction

In cooperative breeders, all group members are capable of reproducing, yet only one dominant female, or one dominant pair, usually produce the majority of the offspring. Hence, most group members, the subordinate helpers, choose to stay and help rearing the offspring of the dominant(s), while foregoing or delaying their own reproduction (Cockburn 1998; Clutton-Brock 2002). However, productivity of social groups commonly follows the law of diminishing returns, whereby each additional group member contributes a smaller increase in group productivity, which means that a subordinate’s efforts might be worth less in larger groups (known as Michener’s Paradox (Michener 1964)). Staying and helping will be favoured by evolution only as long as the average associated benefits exceed those from alternative breeding tactics, such as breeding alone or recruiting others to start a new group (Griffin and West 2003; Leadbeater et al. 2011). Hence, the inclusive fitness payoffs (direct and indirect fitness benefits combined, Hamilton 1964a; Hamilton 1964b) from an individual’s alternative options, relative to those offered by its current group, determine the point at which subordinate helping is no longer advantageous and an individual would benefit from leaving to pursue an alternative breeding tactic (Reeve 1998; Grinsted and Field 2017a). It follows that if group productivity correlates positively with the inclusive fitness payoffs to a subordinate, as tends to be the case in the paper wasp *Polistes dominula* (Leadbeater et al. 2011, Fig. S3), this individual will be better off pursuing alternative options near to the point where its efforts as an extra helper contribute to a negligible increase in group productivity.

*Polistes dominula* is a cooperative breeder that forms small groups of typically fewer than 10 females in early spring at our field sites in Southern Spain (Field and Cant 2007; Field and Leadbeater 2016; Grinsted and Field 2017b). A single dominant female lays most or all of the eggs in the nest, while subordinates forage and build the nest (Reeve 1991; Leadbeater et al. 2011). Subordinates may gain indirect fitness benefits from helping a related dominant (group members are often, but not always, sisters or cousins in *P. dominula*) (Queller et al. 2000; Liebert and Starks 2006; Leadbeater et al. 2010), or direct fitness by either laying a small proportion of the eggs in the nest, or inheriting the dominant breeding position if the dominant dies (Leadbeater et al. 2011). However, in this species, there are high-quality outside options available: subordinates have the possibility to leave and join other groups, or found a new nest alone or with others, if conditions in their nest become unfavorable (Grinsted and Field 2017a; Grinsted and Field 2017b). Both options offer a significant chance of becoming the dominant breeder on their new nest (Grinsted and Field 2017a). The fitness payoffs to dominant breeders, and to subordinates that are genetically related to the dominant, are strongly linked to the productivity of the group and appear to increase with increasing group size throughout the range of commonly observed group sizes (2–15 group members) (Leadbeater et al. 2011, Supporting Online Material). This begs the question: why do *P. dominula* normally form small groups, of on average 5–7 females, rather than giant colonies? Is there a point at which the addition of a new group member contributes so little to group productivity that she has higher payoffs through nesting alone or in another, smaller group? Fitness benefits gained by *P. dominula* subordinates include assured fitness returns (AFR), whereby a subordinate’s investments into increasing colony productivity are not lost if she dies, because the group will rear through the additional brood that resulted from the subordinate’s efforts (Gadagkar 1990; Nonacs 1991; Reeve 1991; Shreeves et al. 2003). However, if a subordinate joins a nest and her efforts result in no increase in colony productivity, i.e. no additional brood produced, there can be no AFR. In theory, larger groups are also expected to fail less often (Nonacs 1991; Reeve 1991), but previous results from our study population have been inconsistent on this point (Shreeves et al. 2003; Leadbeater et al. 2011).

While group size is important, other factors than simply the number of group members also have the potential to influence group productivity. Firstly, because subordinate helpers that are genetically unrelated to the dominant stand to gain less by helping than related helpers, they might be expected to invest less in their groups, by working less hard. However, several studies have included relatedness as a predictor in questions about work effort, and found little evidence that either relatedness between subordinates and dominants, or the average relatedness between group members, affect individual foraging effort, defense behaviour, or aggression (Queller et al. 2000; Leadbeater et al. 2010; Grinsted and Field 2017a; Grinsted and Field 2017b); but see (Leadbeater et al. 2014). Secondly, body size may correlate with quality, with larger individuals being more efficient at task performance (Cervo et al. 2008). Hence, average body size of group members has the potential to influence group productivity. Thirdly, task differentiation among helpers may increase productivity in social animals, as specialized individuals become more efficient at completing their allocated task, such as nest building or foraging (Wilson 1976; Beshers and Fewell 2001). Some eusocial insects show extreme polymorphism which facilitates task specialization (Wilson 1980), while cooperative breeders like *P. dominula* tend not to show consistent variation amongst individuals in morphological traits which correlate with task performance. However, other more subtle individual differences could lead to task differentiation, such as consistent variation in behavioural types (Grinsted et al. 2013; Grinsted and Bacon 2014), developmental stages (Seeley 1982; Settepani et al. 2013) or body size (Nonacs and Reeve 1995). Indeed, Nonacs and Reeve (1995) found that a greater variation in body size among group members within *P. dominula* colonies was positively correlated with relative nest size, indicating that more variation in body size has the potential to increase nest productivity. Asymmetries in body size and developmental stages could additionally increase productivity because group members that are more similar tend to compete more for dominance, reducing investment in productivity. All group members in *P. dominula* are at the same developmental stage in the founding phase, before the first offspring (workers) emerge in early summer, and behaviour is strongly influenced by the rank an individual occupies in the social hierarchy (rather than vice-versa) (Cant and Field 2001; Cant et al. 2006b; Field et al. 2006). Hence, variation in productivity between groups is more likely to be affected by within-colony variation in body size than variation in developmental stage or behavioural type.

We investigated whether a number of factors influence group productivity in *P. dominula* and asked the question: is there a point at which adding a group member makes negligible contributions to group productivity? This question is particularly pertinent in primitively eusocial wasps, where the relationship between group size and productivity is sometimes linear or even accelerating (Shreeves and Field 2002). Specifically, we tested the effect of group size, average body size, the variation in body sizes, and average genetic relatedness amongst group members on nest growth in the founding phase. Nest growth (average number of cells added per day) was used as a proxy for group productivity, as larger nests produce higher numbers of workers, and more workers ensure the production of a higher number of reproductives. Although cells may eventually be re-used, this cannot happen before the first workers mature. We tested to ensure that nest growth was indeed a reliable estimate of group productivity during the pre-worker phase.

## Methods

### Study species and field site

Field work was carried out at our field site in Southern Spain, near Conil de la Frontera, Cadiz (36° 17′ 10.9″ N 6° 03′ 58.1″ W). In early spring at these sites, overwintered, mated females from the same generation found hundreds of nests on stretches of cactus hedges (*Opuntia spp.*) (Leadbeater et al. 2011). During this founding phase, *P. dominula* live as cooperative breeders with a linear dominance hierarchy (Pardi 1948; Cant and Field 2001). The first offspring to mature in late spring become workers on the nest (the worker phase) (Reeve 1991) while the offspring maturing during summer become the reproductives that mate, overwinter and restart the cycle the following spring (Leadbeater et al. 2011). We focused on the growth of nests during the founding phase before the first workers matured. Larger nests produce more workers that can help to rear a larger number of reproductives (Reeve 1991; Cant et al. 2006a; Leadbeater et al. 2011). Hence, nest growth was used as a proxy for colony productivity. Nests were expected to grow in a non-linear fashion, slowing down as larvae matured (Karsai et al. 1996). This might occur because larger larvae require a larger share of the colony resources that cannot then be used to extend the size of the nest by building more cells. We accounted for this in the statistical analyses.

### Data collection

We counted the number of cells in a total of 65 nests in the beginning of the founding phase and again about 1 month later (median 31 days later, range 19 to 45 days) during two field seasons (March through April) in 2013 and 2014. As a measure of nest growth, we used the slope of a linear regression between cell count and number of days, i.e. the average number of cells added per day (range = 0.19 to 1.63; median = 0.81 cells per day).

After nest initiation at the beginning of each season, all wasps on the nests were collected early in the morning (6.00–7.00) and individually marked with a combination of four coloured dots of non-toxic enamel paint applied to the thorax. Group sizes ranged from 2 to 12 females (median = 5). As a measure of body size, wing length was measured to the nearest 0.1 mm using digital calipers. DNA samples were taken by removing the tarsus from a middle leg. Tarsi were kept in 100% ethanol until used for genetic analyses. Wasps were released near to their nests the same morning, before 11.00.

Every 2–4 days, we censused the nests during day time (11.00–17.00), recording which individuals were present. Furthermore, every 10–14 days, we censused the nests early morning (6.00–7.00) before wasps started foraging. If new, unmarked individuals had appeared on a nest, we collected those the following morning for marking, wing measurement and DNA sampling as described above. Morning and day time censuses were used to get a clear picture of which individuals were stable nest residents during the founding phase, to accurately estimate group size, and calculate coefficient of variation in body size (CV = standard deviation/mean), average body size and average relatedness. Colonies had relatively stable group membership throughout the period of nest growth used in this study, and so group sizes did not fluctuate.

Additionally, in a subset of the 65 nests (*N* = 17), we continued censusing until the first workers matured to estimate group productivity (i.e. brood value at worker maturation as in Grinsted and Field (2017a, 2017b)). Brood value was measured by counting the number of cells in the nest, and categorizing the development of brood in each cell as follows: small larvae (given a value of 1.5), medium larvae (2), large larvae (3) and pupae (4); a cell without a larva or pupa was assumed to contain an egg (1).

### Genotyping and relatedness

We followed genotyping protocols described previously (Grinsted and Field 2017b). Briefly, DNA was extracted from tarsus samples and samples were genotyped at nine microsatellite loci used previously in studies of the same population (Strassmann et al. 1997; Henshaw 2000; Leadbeater et al. 2010; Leadbeater et al. 2011; Grinsted and Field 2017a; Grinsted and Field 2017b). All loci were amplified in a single multiplex reaction using the Qiagen multiplex PCR kit (Qiagen, Venlo, The Netherlands). Relatedness 5.0.8 software (Queller and Goodnight 1989) was used to calculate relatedness between joiners and group members as in Grinsted and Field (2017b).

### Statistics

All statistical analyses were performed in R (R Core Team 2016). Our main model was a linear model (LM) with a Gaussian error structure that tested the effect of various predictors on nest growth (*N nests* = 65). As response variable, we used the slope of nest growth (i.e. the average number of cells added to the nest per day) in the observation period. The slopes were square root transformed to meet the assumptions of homogenous and normally distributed residuals. The following predictor variables were included in the LM: colony size (i.e. number of group members), colony-size^2 to allow for a non-linear effect of colony size on nest growth, the average relatedness among group members, the average wing length of group members, the number of cells on the first observation date (first cell count) and the date of the first observation (first observation date). The latter two factors were included to control for the effect of potential non-linear nest growth: starting observations at different points in a non-linear growth curve will affect the slope of a linear regression. A nest that was relatively large on the first day of observation may be nearer to its plateau in growth, resulting in a negative effect of first cell count on the slope of nest growth. A nest that was founded slightly later in the season, leading to a later first observation date, would be observed for a shorter period of time and be observed in the steeper part of its growth curve, resulting in a positive effect of first observation date on the slope of nest growth. Predictors with no effect on the response variable (*p* > 0.10) were omitted from the LM to obtain more reliable *p* values for the remaining predictors.

From the slope of nest growth values predicted by the main model for each group size (group sizes 2 to 12; first cell count and first observation date set to their median values), we calculated the predicted increase in nest growth expected by adding one extra group member (*predicted increase in nest growth for group size N = predicted nest growth value for group size N + 1—predicted nest growth value for group size N*). We further calculated the percentage increase in nest growth predicted through adding one extra group member (*predicted percentage increase for group size N* = *predicted increase in nest growth for group size N/predicted nest growth value for group size N* × *100*).

To test whether variation in body sizes within groups affects nest growth, we ran the same model but replacing average wing length with CV wing length. Average wing length and CV wing length could not be included in the same model because they were significantly correlated (*Pearson’s r* = − 0.36, *p* = 0.0029). The *p* values for the other predictors were qualitatively similar in both models, and only the effect of CV from this model is reported in the results section.

Finally, we tested the assumption that nest growth was a reliable measure of group productivity by correlating the slope of nest growth with brood value at the time the first workers matured in a subset of 17 nests.

#### Data availability

All data generated and analyzed during this study are included in this paper’s online supplementary information (Online Resource 1).

## Results

**Fig. 1:**
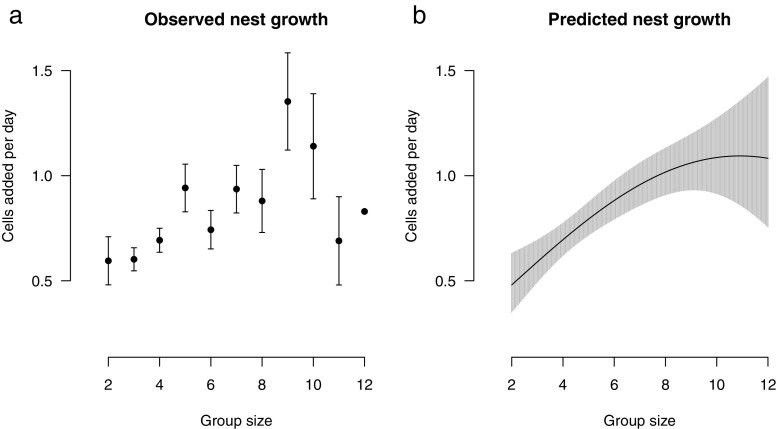
Nest growth measured as the average number of cells added per day plotted against group size (number of group members). **a** Average observed nest growth with standard errors (except for *group size* = 12 because *N* = 1). **b** Predicted nest growth based on the main model (model: nest growth = group size + group size^2 + median first cell count + median first observation date); shaded area represents 95% confidence bands

Group size was a significant, positive predictor of nest growth (*p* = 0.0074; Table 1; Fig. 1a,b), while average relatedness among group members, average wing length of group members and CV wing length did not predict nest growth (each predictor *p* > 0.30; Table 1). As predicted, if nests grow in a non-linear fashion, number of cells at first observation had a negative effect on nest growth (*p* < 0.0001) and date of first observation had a positive effect (*p* = 0.036).

**Table 1:** Results from the main model testing the effect of various predictors on the response variable nest growth (number of cells added per day)

Response variable: Nest growth (average number of cells added per day)		
Predictor variable	*t* value	*p* value
Group size	2.77	0.0074
Group size^2	− 1.68	0.098
Number of cells at first observation	− 5.59	< 0.0001
Date of first observation	2.15	0.036
Average genetic relatedness	0.76	0.45
Average wing length	1.03	0.31
Coefficient of variation in wing length	0.14	0.89

There was a marginally non-significant trend for the quadratic term of group size (group size^2) to have a negative effect on nest growth (*p* = 0.098) and it was therefore retained in the model. Inspecting the raw data clearly suggests that the effect of group size on nest growth may be non-linear in nature, and the reason why the term only reaches near-significance is likely due to the smaller sample sizes of larger nests where the effect plateaus: group sizes of > 8 are less abundant in the population (in our sample *N nests of 1–8 residents* = 57; *N nests of 9–12 residents* = 8).

**Fig. 2:**
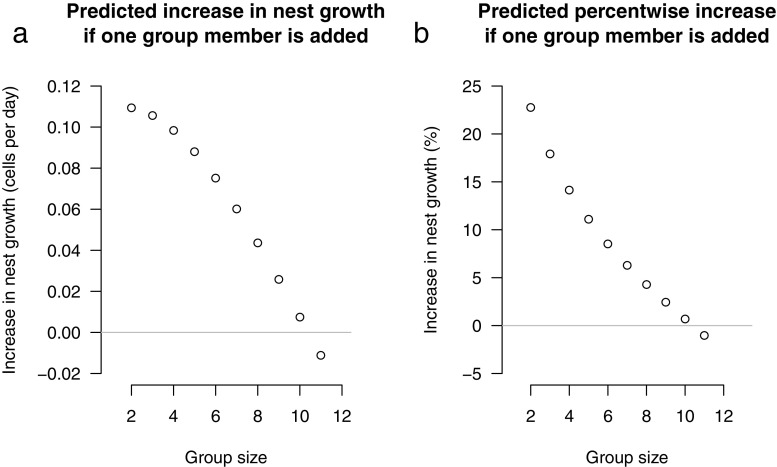
Predicted increase (**a**) and predicted percentwise increase (**b**) in nest growth based on the main model (model: nest growth = group size + group size^2 + median first cell count + median first observation date) plotted against group size; e.g. the point at *group size* = 2 represents the increase if one extra wasp was added to make a group of 3. The grey line indicates the point at which adding extra group members no longer translates to an increase in nest growth

Both the predicted increase in nest growth (Fig. 2a) and the predicted percentwise increase in nest growth (Fig. 2b) based on the parameter values from the main model decreased with group size and became negative for groups larger than 10: by adding an extra group member, a group of two wasps had the greatest predicted increase in nest growth (0.11 extra cells added per day equivalent to 22.76% extra nest growth) while a group of 10 had a close-to-zero predicted increase (0.0074 extra cells added = 0.68%) and a group of 11 had a predicted *decrease* in nest growth (− 0.011 extra cells added = − 1.02%; Fig. 2).

**Fig. 3:**
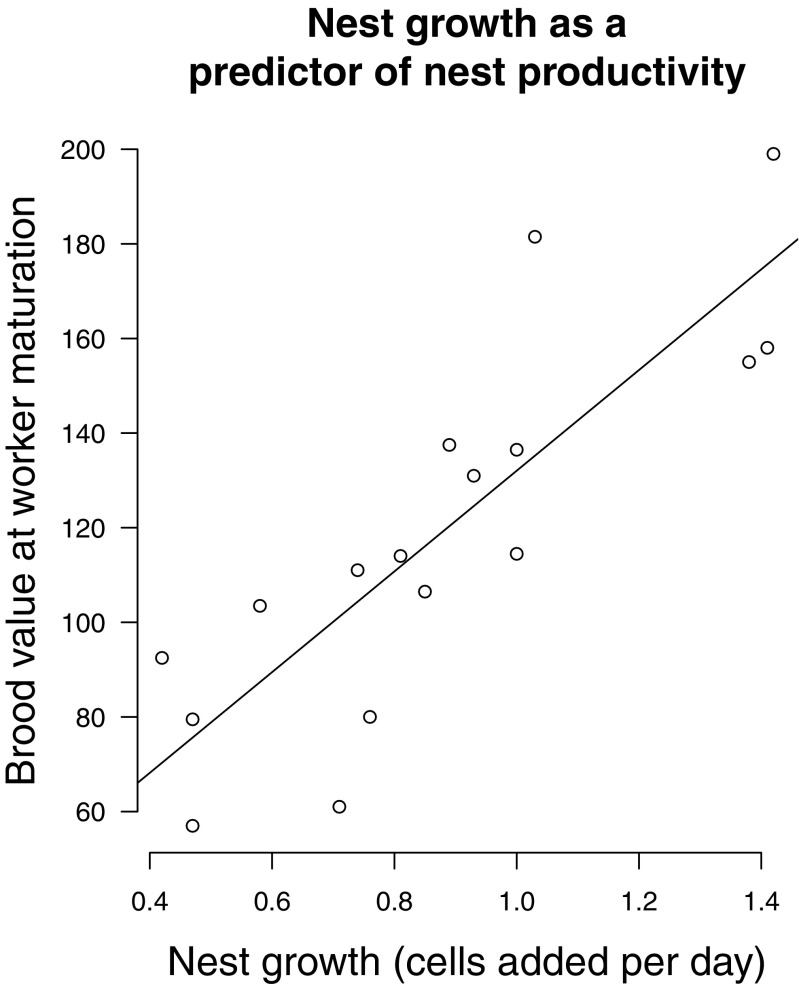
Brood value (egg = 1, small larva = 1.5, medium larva = 2, large larva = 3, pupa = 4) at the time when the first worker(s) matured, as a measure of nest productivity, plotted against nest growth (number of cells added per day) in a subset of 17 nests. The full line is a regression line indicating a significant correlation (LM, *p* < 0.0001)

Nest growth was a reliable predictor of nest productivity: we found a highly significant, positive correlation between brood value at worker maturation and nest growth (Fig. 3; LM, *t* = 5.85, *p* < 0.0001).

## Discussion

We tested the effect of various predictors on nest growth—a proxy for group productivity—in *P. dominula* during the founding phase, and found group size to be the only significant predictor. Importantly, group size had a non-linear effect on nest growth, such that the advantage of increasing the size of the group was highest for smaller groups. Once reaching a group size of 10, additional group members contributed negligibly to nest growth (Fig. 2; see also Wenzel (1996) in *Polistes annularis*). Although body size has been suggested to be an indicator of individual quality in *P. dominula* (Cervo et al. 2008), we found no evidence that average body size affected nest growth. Body size tends to be a predictor of whether an individual becomes a dominant breeder or a subordinate helper, with larger females becoming dominants (Nonacs and Reeve 1995), but our results suggest that groups comprising larger individuals are not more productive. Furthermore, our results did not support the hypothesis that greater variation among the body sizes within a group results in greater nest growth (Nonacs and Reeve 1995), which might have been expected if there was task specialization based on body size variation in *P. dominula*, or if body size asymmetries within groups reduce levels of conflict. Genetic relatedness between group members also did not affect nest growth, corroborating previous findings that relatedness has little influence on group productivity, individual investment in the nest, or reproductive skew in this species (Queller et al. 2000; Liebert and Starks 2006; Leadbeater et al. 2010; Grinsted and Field 2017a; Grinsted and Field 2017b). Nest growth during the founding phase represents only one component of lifetime fitness. Other important fitness components will include, for example, group survival and the number of reproductives produced towards the end of the colony cycle, both of which are expected to increase with group size (Nonacs 1991; Leadbeater et al. 2011).

The optimal group size for a cooperative breeder depends on the costs and benefits associated with group living. High failure rates and low productivity of solitary breeders in *P. dominula* (> 90% of nests founded by single females fail) (Nonacs and Reeve 1995; Nonacs et al. 2006; Leadbeater et al. 2011; Zanette and Field 2011) result in higher average direct fitness payoffs through group living for both dominants and subordinates in our study population, whether subordinates are related or unrelated to the dominant (Leadbeater et al. 2011). Assured fitness returns ensure that investments into the nest are not wasted should a group member die, whereas all efforts are wasted if a lone breeder dies before maturation of her offspring (Gadagkar 1990; Nonacs 1991; Reeve 1991; Shreeves et al. 2003). These previous results explain why most *P. dominula* foundresses form groups at our Spanish field sites, rather than attempt to breed alone, but do not explain why groups rarely exceed 10 foundresses. Leadbeater et al. (2011) found that from the perspective of the dominant and any helpers related to her (sisters and cousins), inclusive fitness payoffs increase virtually exponentially with group size. However, *P. dominula* is probably not capable of true kin recognition (Queller et al. 2000; Leadbeater et al. 2010; Grinsted and Field 2017b); but see (Leadbeater et al. 2014), preventing foundresses from forming giant colonies comprising only close relatives such as full sisters, and females commonly join nests containing no sisters despite having sisters in nests nearby (Grinsted and Field 2017a). From the perspective of unrelated helpers, Leadbeater et al. (2011) found that direct fitness through nest inheritance combined with subordinate egg-laying peaks around a group size of eight or nine, and drops to close to zero for larger groups (Leadbeater et al. 2011, Supporting Online Material). Since direct fitness is the only potential  payoff for an unrelated helper, non-relatives may gain little advantage from staying and helping in groups larger than that. Interestingly, we similarly identified a group size of 10 as the point at which the addition of extra group members starts to contribute negligibly to nest growth. Whether or not there is a causal relationship between the two factors remains to be tested, but it seems likely that both contribute to the apparent optimal group size of < 10 at our field site.

Michener (1964) first reported that larger groups of social Hymenopterans had lower per-capita productivity (known now as Michener’s paradox). However, the proximate mechanisms behind this phenomenon have yet to be elucidated (see Wenzel and Pickering (1991) for a possible ultimate explanation), and linear or even accelerating relationships have previously been found in primitively eusocial wasps (Shreeves and Field 2002). There may be several proximate explanations as to why the addition of a group member contributes less to nest growth in larger groups of *P. dominula*. If the cause of reduced per-capita nest growth is reduced per-capita work efforts, it may either be a result of a new group member working less hard than the average resident subordinate, or it may occur because a new arrival causes existing residents to reduce their work efforts. There is no evidence for the former hypothesis: new joiners in fact appeared to work harder, rather than less hard, than resident subordinates on their new nests in Grinsted and Field (2017a). There is, however, good evidence for the latter hypothesis: individuals of a given rank typically work less hard in larger groups (Cant and Field 2001; Field et al. 2006; Leadbeater et al. 2010). Additionally, work efforts might not have a linear, additive effect on nest growth because of an impediment on nest growth in larger colonies. This impediment could result from factors such as limits in the ability of the dominant to produce offspring at the maximum possible rate of nest growth, increased competition for limited resources such as nesting material and forage or heightened within-group aggression. It is unlikely that nest growth is limited by the dominant’s ability to produce offspring: Mead et al. (1994) report an average of 2.5 eggs laid per day in *P. dominula* nests which easily exceeds the number of new cells added per day in this study (range = 0.19 to 1.63 cells per day). It is also hard to imagine how competition for resources in the environment would be higher when a wasp joins the group, compared with the typical alternative of her joining a neighboring group instead (Grinsted and Field 2017a): in either case, she remains in the environment. While *P. dominula* aggression rates were not affected by group size in Cant et al. (2006b), conflicts did tend to escalate more in larger groups in Cant et al. (2006a). An interesting avenue for further studies will therefore be to identify whether greater conflict and aggression might inhibit nest growth, and therefore group productivity, in larger colonies.

## Electronic supplementary material

Online Resource 1 Excel data sheet with the raw data used for the analyses presented in this paper (XLSX 18 kb)
